# Graphite carbon-encapsulated metal nanoparticles derived from Prussian blue analogs growing on natural loofa as cathode materials for rechargeable aluminum-ion batteries

**DOI:** 10.1038/s41598-019-50154-8

**Published:** 2019-09-20

**Authors:** Kaiqiang Zhang, Tae Hyung Lee, Bailey Bubach, Ho Won Jang, Mehdi Ostadhassan, Ji-Won Choi, Mohammadreza Shokouhimehr

**Affiliations:** 1 Department of Materials Science and Engineering, Research Institute of Advanced Materials, Seoul National University, Seoul, 08826 Republic of Korea; 2 Electronic Materials Center, Korea Institute of Science and Technology (KIST), Seoul, 136-791 South Korea; 3 Department of Petroleum Engineering, University of North Dakota, Grand Forks, ND 58202 United States

**Keywords:** Batteries, Energy

## Abstract

Aluminum-ion batteries (AIBs) are attracting increasing attention as a potential energy storage system owing to the abundance of Al sources and high charge density of Al^3+^. However, suitable cathode materials to further advance high-performing AIBs are unavailable. Therefore, we demonstrated the compatibility of elemental metal nanoparticles (NPs) as cathode materials for AIBs. Three types of metal NPs (Co@C, Fe@C, CoFe@C) were formed by *in-situ* growing Prussian blue analogs (PBAs, Co[Co(CN)_6_], Fe[Fe(CN)_6_] and Co[Fe(CN)_6_]) on a natural loofa (L) by a room-temperature wet chemical method in aqueous bath, followed by a carbonization process. The employed L effectively formed graphite C-encapsulated metal NPs after heat treatment. The discharge capacity of CoFe@C was superior (372 mAh g^−1^) than others (103 mAh g^−1^ for Co@C and 75 mAh g^−1^ for Fe@C). The novel design results in CoFe@C with an outstanding long-term charge/discharge cycling performance (over 1,000 cycles) with a Coulombic efficiency of 94.1%. *Ex-situ* X-ray diffraction study indicates these metal NP capacities are achieved through a solid-state diffusion-limited Al storage process. This novel design for cathode materials is highly significant for the further development of advanced AIBs in the future.

## Introduction

With the gradual reduction of fossil energy resources, increasing environmental problems, and increasing demand for energy, the search for an ideal energy has become highly challenging^1^. Currently, integrating clean, sustainable energy resources (solar, wind, geothermal, *etc*.) into the electric grid is considered as a potential solution^2^. However, their intermittencies are major obstacles that require energy storage devices. Hence, batteries have received significant attention and are considered as an ideal candidate owing to their unique energy storage mechanism (electrical energy to chemical energy). Meanwhile, the vigorous development of such devices can be applied to the electric energy storage in a power grid, as well as realize the transformation of the current traction of internal combustion engines to the fully electric traction vehicles, which will improve the urban environmental quality significantly^3^. Hitherto, lithium-ion batteries have demonstrated great success. However, cost is a primary obstacle hindering the mass application of lithium-ion batteries^4^. Hence, scientists are attempting to develop other low-cost metal ion batteries (*e.g*., Na-, K-, and Al-ion batteries)^5–7^.

Aluminum ranks first in metal content in the Earth’s crust. Therefore, aluminum ion batteries (AIBs) are ideal for solving cost problems. In addition, Al possesses competitive volumetric and gravimetric capacities (2978 mAh g^−1^ and 8034 mAh cm^−3^, respectively) to Li anodes (3870 mAh g^−1^ and 2080 mAh cm^−3^, respectively)^8^. Research on aluminum-ion batteries has increased in the last five years, primarily focusing on the development of cathode materials to overcome the higher charge density of Al^3+^ or large ionic radius of AlCl_4_^−^ ^9^. Currently, the research on cathode materials of aluminum batteries focuses primarily on carbon- and sulfide-based cathode materials that demonstrate significant improvements in performance^10–13^. In brief, the present research on cathode materials has focused on developing new compounds or unique material structures. This has inspired us to investigate elemental metal nanoparticles (NPs) as cathode materials for AIBs. To obtain such NPs, a medium for dispersing metal NPs is necessary to inhibit infaust agglomerations. Meanwhile, an effective protective layer for isolating metal NPs and the ambient environment is essential, as metal NPs are generally active. A three-dimensional (3D) network material is an ideal structure option for dispersing metal NPs; however, the reported 3D foam is currently widely used as a current collector (e.g., nickel and copper foams, *etc*.)^14,15^, and is not appropriate in this study requiring a nonmetallic 3D interconnected framework. In the search for such a 3D material, we found loofa (L) from nature. L is a natural nonmetallic 3D framework-like material with extremely low cost and no pollution.

Two primary approaches can be used for the metal NP loading process: i. direct chemical reduction of the metal ions to the surface of L (i.e. wet chemical method); ii. heat treatment of the precursor in a reducing atmosphere. It is noteworthy that L exhibits a natural carbonaceous feature that requires heat treatment for carbonization in the subsequent preparation process of electrode materials. Hence, the second option is more appropriate and interesting. More importantly, a highly crystallized and defect-free carbon matrix requires a high temperature of up to 3000 °C for heat treatment^16^. However, in the presence of metal NPs, the crystallization temperature of carbon is reduced significantly (less than 1000 °C)^17,18^. Therefore, carbon species are expected to crystallize spontaneously on the surface of metal NPs at a relatively low temperature to, *in situ*, form a graphite C layer for the protection of the metal NPs.

The remaining effort is to obtain a suitable process and material to grow the precursor of the metal NPs on the L surface. The presence of metallic elements in the precursor has inspired us to consider the currently popular metal-organic frameworks (MOFs). MOFs are promising candidates for linking nonmetallic L and metal ions owing to their special organic bridges. Among the MOFs, Prussian blue analogues (PBAs) exhibit the advantage of rich-C, simple synthesis, and easy mass production at room temperature by a facile wet chemistry method in aqueous base^19,20^.

In this study, Fe[Fe(CN)_6_] (FeHCFe), Co[Co(CN)_6_] (CoHCCo), and Co[Fe(CN)_6_] (CoHCFe) as precursors were loaded on the L surface, followed by heat treatment under reducing air (see experimental section for more detailed information). The formed elemental metal NPs encapsulated by crystallized carbon (Co@C, Fe@C, and CoFe@C) served as cathode materials for the AIBs, indicating a solid-state diffusion-controlled activity toward Al storage.

## Methods

### Electrode material synthesis

L was collected from a farm near our campus and washed several times using deionized (DI) water; 0.01 M of two CoCl_2_ and one FeCl_3_ solutions were prepared in three beakers. Subsequently, the well-cleaned L was soaked into these three solutions. Meanwhile, 0.01 M of one K_3_Co(CN)_6_ and two K_3_Fe(CN)_6_ solutions were prepared in another three beakers. The solutions mentioned herein were prepared using DI water without any organic additives. After overnight soaking the L in these three salt solutions, K_3_Co(CN)_6_ was added to the CoCl_3_ solution, and the two K_3_Fe(CN)_6_ solutions were added into the FeCl_3_ and CoCl_3_ solutions, to *in-situ* grow CoHCCo, FeHCFe, and CoHCFe on the L surface. To avoid the aggregation of Prussian blue analog NPs, the adding procedure was performed dropwise. These mixed solutions were constantly stirred overnight, after which the soaked L was removed followed by rinsing with DI water to remove the loosely attached Prussian blue analog NPs.

The L loaded with CoHCCo, FeHCFe, and CoHCFe was dried in a vacuum oven followed by heat treatment in Ar/N_2_ (96/4%) atmosphere at 700 and 900 °C for 1 and 5 h, respectively. The carbonized loofa loaded with metal NPs (Co@C, Fe@C, and CoFe@C) were collected for subsequent characterizations.

### Characterizations

The morphologies of the prepared CoHCCo, FeHCFe, CoHCFe, Co@C, Fe@C, and CoFe@C were investigated through field emission-scanning electron microscopy (FE-SEM, Inspect F50) and transmission electron microscopy (TEM, Tecnai F20). Consistent elements of the as-prepared products were qualitatively detected with energy-dispersive X-ray spectroscopy (EDX). A structural study and phase verification were performed through X-ray diffraction (XRD, D8-Advance, fixed incident angle of 2°, equipped with Cu Ka radiation). The crystallinities of C derived from the L were measured through Raman spectroscopy (inVia Raman Microscope). The bonding features of C and metal NPs were detected through X-ray photoelectron spectroscopy (XPS, PHI 5000 VersaProbe) using an Al Kα source (Sigma probe, VG Scientifics). The thermal stability of the as-prepared carbonized L was investigated through thermogravimetric analysis (TGA) that was performed under an air flow from room temperature to 700 °C with a temperature ramp of 10 °C min^−1^. Inductively coupled plasma (ICP) was used for the quantitative measurement of the amount of metallic element in each sample. The ICP samples were prepared by dissolving Co@C, Fe@C, and CoFe@C into aqua regia solutions, followed by ICP measurements. Mesoporous hollows were examined by N_2_ gas Brunauer-Emmett-Teller (BET) adsorption-desorption isotherms.

### Electrochemical property

The as-prepared Co@C, Fe@C, and CoFe@C were ground with super P and polyvinylidene fluoride in a mass ratio of 7:2:1, after which the mixed powders were dispersed into a constantly stirred N-methyl-2-pyrolidinone solution to prepare a uniform slurry for subsequent electrochemical characterizations. The well-prepared slurry was cast on an Pt deposited organic polymer (~3 mg cm^−2^) by referring other literature^21^ and dried in a vacuum oven at 80 °C overnight to form the final cathode of the AIBs.

The electrochemical properties of Co@C, Fe@C, and CoFe@C were characterized in the pouch cells where the well-dried electrodes were inserted as the cathodes, and Al metal foil (0.5 mm in thickness) was used as the anode. Between the two electrodes, two pieces of glass-fiber papers (Whatman) soaked with 1-Ethyl-3-methylimidazolium chloride ([EMIM]Cl)/AlCl_3_ (1/1.3 mole/mole) was inserted to isolate the anode from the cathode.

The electrochemical dynamic performance was characterized with electrochemical impedance spectroscopy (EIS, Im6ex ZAHNER) in the assembled pouch cell with a frequency range of 10 mHz to 1 MHz and a voltage amplitude of 10 mV.

Cyclic voltammetry (CV) measurements were performed in the potential range of 0.05–1.2 V vs. AlCl_4_^−^/Al with a scan rate of 0.5 mV s^−1^ (WBCS3000, Wonatech, Korea). Galvanostatic charge/discharge cycling measurement was performed between 0.05–1.2 V vs. AlCl_4_^−^/Al at various current densities corresponding to 100 and 1,000 mA g^−1^. A long-term lifetime measurement was conducted at the current density of 1,000 mA g^−1^. The current densities and specific capacities herein were calculated based on the weight of the metallic active materials.

### *Ex-situ* characterization

The samples for *ex-situ* XRD characterizations were prepared by disassembling the pouch cells charged/discharged to 1.2/0.05 V vs. AlCl_4_^−^/Al, followed by rinsing with sufficient ethanol and drying in a vacuum oven.

## Results and Discussion

**Figure 1:**
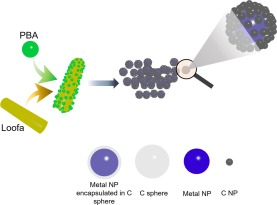
Synthesis procedure of the C encapsulated metal NPs.

**Figure 2:**
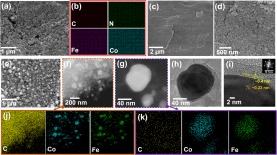
(**a**) SEM image and (**b**) EDX mapping of CoHCFe. SEM images of (**c**) well-cleaned loofa surface; (**d**) *in-situ* grown CoHCFe on the loofa surface. (**e–h**) SEM, STEM, magnified STEM, and TEM images of CoFe@C. (**i**) HRTEM of the crystallized CoFe alloy and C layer on the surface. (**j,k**) EDX mapping of CoFe@C. The CoFe@C was prepared through heating at 900 °C for 5 h.

The synthesis procedure of the innovative products was illustrated in Fig. 1. Sphere-shaped FeHCCo NPs of less than 100 nm were exhibited in the SEM images (Fig. 2a). Similar sphere-shaped NPs for CoHCCo are exhibited in Supplementary Fig. S1a, and are different from the cubic-shaped FeHCFe (Supplementary Fig. S2a). Furthermore, the uniform distribution of the consistent elements for each of the NPs were further confirmed by EDX mapping (Fig. 2b, Supplementary Figs S1 and S2).

The employed natural L foam displays a ribbon-like 3D structure with rich internal channels representing a highly improved specific surface area (Supplementary Fig. S3). A high specific area is important for the mass loading of CoHCCo, FeHCFe, and CoHCFe. After the *in-situ* growth of CoHCCo, FeHCFe, and CoHCFe, a significant difference in the L surface is observed (Fig. 2c,d, Supplementary S4). The surface of the L was fully covered with the *in-situ* grown NPs. In addition, the interior of the channels in each L ribbon was well loaded, although a slight reduction in the loading amount was shown (Supplementary Figs S4 and S5). Carbonization is considered as a key procedure to obtain final electrode materials with a well-constructed porous physical structure. In this study, we performed four types of heat-treatment conditions (700 and 900 °C for 1 and 5 h, respectively). Samples annealed at 700 °C for 1 and 5 h exhibit a clogged sponge-like structure (Supplementary Fig. S6a,b). However, some residual species were observed for the CoHCCo-loaded L when treated at 900 °C for 1 h (Supplementary Fig. S6c). After the final optimization for the heat-treatment condition (at 900 °C for 5 h), a sponge-like porous C matrix decorated with metal NPs was observed (Supplementary Fig. S6d). The same heat-treatment conditions can be obtained for another Fe@C and CoFe@C (Supplementary Figs S7 and S8). The uniformly distributed metal NPs are also demonstrated by the SEM image of a representative CoFe@C (Fig. 2e) and scanning transmission electron microscopy (STEM, Fig. 2f), where small metal NPs mired in the C matrix are apparent. The porous C matrix disperses the metal NPs effectively, thus limiting the particle size to ~70 nm (Fig. 2g,h).

Such small NPs were expected to enhance the contact surface area with infiltrated electrolytes, facilitating the improvement in use efficiency of the active materials. Magnified STEM and TEM images (Fig. 2g,h) clearly exhibit that the carbon layers cover the surface of the metal NPs to form a CoFe@C structure. To verify the crystallized condition of C as described in the introduction section, high-resolution TEM (Fig. 2i) displays the C lattice planes (~15 layers) with an interplanar spacing of ~0.4 nm; this is different from the inside metal NPs with a much smaller interplanar spacing of ~0.22 nm. This highly crystallized feature was also verified by the fast Fourier transform pattern, in which a single crystal diffraction pattern was clearly observed. The consistent elements for the specially designed materials were detected with EDX mapping (Fig. 2j,k and Supplementary Fig. S9). The Co, Fe, and C elements were clearly detected in both high and low magnifications, revealing the alloy matrix of the NPs. Similar elemental constitutions can be found in other samples of Co@C and Fe@C (Supplementary Figs S10 and S11). This *in-situ* grown carbon layer was expected to protect the integrity of the metal NPs to exhibit an outstanding lifespan^22^.

**Figure 3:**
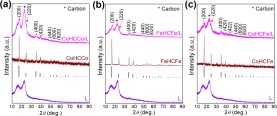
XRD diffraction patterns of (**a**) L, CoHCCo, and CoHCCo/L, (**b**) L, FeHCFe, FeHCFe/L, (**c**) L, CoHCFe, and CoHCFe/L.

**Figure 4:**
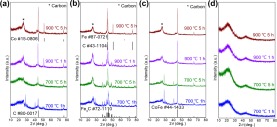
XRD spectra of carbonized L loaded with (**a**) CoHCCo, (**b**) FeHCFe, and (**c**) CoHCFe at various parameters. (**d**) Heat-treated naked L without any loading.

The phase variation during the synthesis process was examined with X-ray diffraction (XRD, Fig. 3). The characteristic peaks of the CoHCCo, FeHCFe, and CoHCFe were well indexed after *in-situ* growing on the L surface, demonstrating that the cyanide organic linkers can easily coordinate with L at room temperature in aqueous bath. The generated phase for each sample by heat treatment are shown in Fig. 4, where C/metal compounds (e.g., Fe_3_C) were observed when the annealing temperature was set at 700 °C. However, CoHCCo/L and CoHCFe/L exhibit a separated phase, i.e. C and elemental metal, although a Fe_3_C phase was shown for FeHCFe/L, when heated at 900 °C for 1 h. In addition, the crystallization of C was slightly improved after carbonization at 900 °C compared to that at 700 °C. We thus employed the parameters (900 °C, 5 h) for subsequent heat treatments. It is not necessary to further enhance the temperature and prolong the processing time as the elemental metals have been obtained. The crystallized C as shown in the TEM image (Fig. 2i) was further demonstrated by XRD (Fig. 4a–c), where a sharp (002) peak of C was indexed in the samples with metal NPs encapsulated inside; nevertheless, the naked L after heating displays only an amorphous C hump (Fig. 4d).

**Figure 5:**
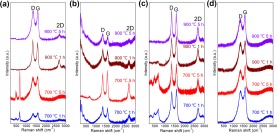
Raman spectra of **(a**) Co@C, (**b**) Fe@C, (**c**) CoFe@C, and (**d**) L prepared at diverse carbonization conditions.

The characteristics of C were further studied with Raman spectra (Fig. 5), where the D-band (A_1g_ symmetry) caused by the presence of the disordered structure was exhibited at ~1350 cm^−1^ ^23^. The typical G-band at ~1600 cm^−1^ corresponds to the stretching of the C–C bond (E_2g_ symmetry), and it is typical to all sp^2^ carbon systems^24^. However, a significant difference in the Raman spectra of L with and without metal NPs is the 2D band corresponding to a graphitic sp^2^ mode, which further supported the crystallized C as discussed in the XRD and TEM results (Figs 2 and 4)^25–27^. This behavior is absent in the naked L after heat treatment (Fig. 5d), revealing the disordered C matrix. Several uncertain peaks were observed at wavenumbers less than 800 cm^−1^ in the Raman spectra (Fig. 5a–c), and were more likely to be the reflections of oxidized metal NPs at the metal NPs/C interfaces owing to the electron transfer from metal NPs to C^28,29^.

The thermal stabilities of the as-prepared Fe@C, Co@C, and CoFe@C were measured using TGA (Supplementary Fig. S12). A slight weight decrease in the initial range owing to the evaporation of water molecules was indicated until ~400 °C, where a rapid decrease is resulted from the C burning, emphasizing the adequate reliability of the products when served at room temperature. Consequently, residues of ~40% for Co@C and CoFe@C, and of ~30% for Fe@C had remained.

**Figure 6:**
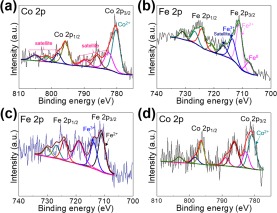
DeconvolutedXPS spectra of (**a**) Co 2p for Co@C, (**b**) Fe 2p for Fe@C, (**c**) Fe 2p and (**d**) Co 2p for CoFe@C.

The surface chemical properties of Co@C, Fe@C, and CoFe@C were studied using XPS. A wide survey scan (Supplementary Fig. S13a–c) for these samples further demonstrated the consistent elements and bonding nature. The deconvoluted Co 2p and Fe 2p XPS spectra (Fig. 6) with relatively lower intensities compared with C 1s (Supplementary Fig. S13d–f) confirm the wrapping layer on the surface of the metal NPs, based on the surface chemical characterization feature of XPS, as observed in the SEM and TEM images (Fig. 2g–i). A further analysis of the deconvoluted Fe 2p and Co 2p exhibits oxidized Co and Fe (Fig. 6) caused by the electron transfer from the metal element to the surrounding crystallized C wrapping layer, although elemental iron was detected in Fe@C (Fig. 6b). However, metal elementals (Co, Fe, and CoFe alloy) being dominated phases are confirmed according to the XRD diffraction results (Fig. 4), indicating that only a small amount of surficial metal atoms bonded with C to promote C crystallization. Thus, it is rational to conclude that the active materials in these composite materials are elemental metal NPs.

A quantitative analysis of the metal elements in Co@C, Fe@C, and CoFe@C were performed with ICP. Co@C consists of 13 wt.% Co; Fe@C consists of 22 wt.% Fe; CoFe@C consists of 12 wt.% CoFe in the basis of exposed samples. The porosity and surface area of the representative CoFe@C were measured with BET. Consequently, the typical adsorption-desorption isotherm curve is depicted (Supplementary Fig. S14). CoFe@C possesses a surface area of 189 m^2^ g^−1^. In addition, the pore size distribution of CoFe@C exhibits concomitant micro- and meso-pores with an average pore radius of ~3.6 nm and volume of ~0.128 cm^3^ g^−1^ calculated from the Barrete-Joyner-Halenda (BJH) desorption.

**Figure 7:**
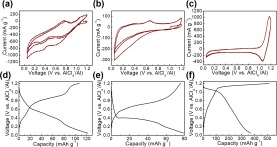
(**a–c**) CV curves and (**d–f**) corresponding charge/discharge voltage profiles of Co@C, Fe@C, and CoFe@C.

The electrochemical properties of Co@C, Fe@C, and CoFe@C were measured in pouch cells, and exhibited an open circuit voltage (OCV) of ~1 V vs. AlCl_4_^−^/Al (Supplementary Fig. S15). The electrochemical activities of Co@C, Fe@C, and CoFe@C were investigated using CV curves at a scan rate of 0.5 mV s^−1^. The CV curve of Co@C (Fig. 7a) shows evident discharge peaks at ~0.6 and 0.3 V vs. AlCl_4_^−^/Al, and accordingly, a charge peak at ~0.8 V vs. AlCl_4_^−^/Al. In addition, the CV curve of Fe@C (Fig. 7b) exhibits a discharge and charge hump at 0.39 and 0.66 V vs. AlCl_4_^−^/Al, respectively. However, the CoFe@C displays a pair of redox peaks at 1.17 V vs. AlCl_4_^−^/Al (charge process) and 1.04 V vs. AlCl_4_^−^/Al (discharge process) (Fig. 7c). A further discussion on the relevant effective electrochemical reactions are provided in the following context. To the best of our knowledge, this is the first example where metal NPs as cathode materials in AIBs exhibit distinct redox peaks, suggesting the electrochemical activity toward Al storage. Furthermore, the potential window of 0.05–1.2 V vs. AlCl_4_^−^/Al is suitable for subsequent characterizations without obvious decomposition behaviors. In a controlled experiment, we evaluated the error that may be introduced by C, although the electrochemical active potential of C was reported to be ~2 V vs. AlCl_4_^−^/Al^30^. No redox peaks were observed in the CV curve of naked C derived from the L, indicating its negligible electrochemical activity (Supplementary Fig. S16a). Accordingly, a negligibly small capacity was observed for C (Supplementary Fig. S16b,c).

**Figure 8:**
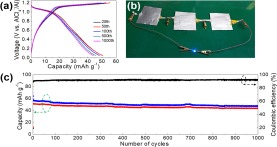
(**a**) Representative charge/discharge voltage profiles of CoFe@C at 1,000 mA g^−1^, (**b**) assembled pouch cells with CoFe@C as cathode material lighting a blue LED lamp, and (**c**) long-term repeated charge/discharge cycling measurement of CoFe@C at 1,000 mA g^−1^.

Galvanostatic charge/discharge was performed at a current density of 100 mA g^−1^ for the three samples. The representative voltage profiles of Co@C and Fe@C (Fig. 7d,e) exhibit discharge plateaus at ~0.5 V vs. AlCl_4_^−^/Al for Co@C and ~0.4 V vs. AlCl_4_^−^/Al for Fe@C, indicating typical electrochemical reaction processes. However, we found that the discharge plateau (~1.1 V vs. AlCl_4_^−^/Al) of CoFe@C was much higher than those of Co@C and Fe@C, which facilitates the enhancement in energy density. The cycling measurements for Co@C and Fe@C as the cathode materials of AIBs were performed at a current density of 100 mA g^−1^ and exhibited stable charge/discharge cycling. Competitive capacities of 103 mAh g^−1^ for Co@C and 75 mAh g^−1^ for Fe@C at the 100^th^ cycle were obtained with Coulombic efficiencies of 95.5% and 99.7%, respectively, (Supplementary Fig. S17a,b). However, compare with other, a 3X higher discharge capacity (372 mAh g^−1^) for CoFe@C was obtained with an undesirable Coulombic efficiency (72%). We thus conducted repeated charge/discharge cycling tests at an enhanced current density (1,000 mA g^−1^) for Co@C, Fe@C and CoFe@C. As depicted in Fig. 8a, stable discharge plateaus during the long-term cycling test were observed for CoFe@C. Practical pouch-type AIBs assembled with CoFe@C-based cathode materials can lighten a 3-V blue LED (Fig. 8b). As the test progressed, a capacity of 44 mAh g^−1^ was maintained with a Coulombic efficiency of 94.1% achieved at the 1000^th^ cycle at a capacity loss of 0.7% in each cycle based on the initial charge capacity of 51 mAh g^−1^, demonstrating the outstanding long life-span performance of CoFe@C as a cathode material for AIBs. However, decreased capacities of 34 mAh g^−1^ for Co@C and 27 mA g^−1^ for Fe@C are obtained at the 1000^th^ cycle. The capacity values are lower than the estimated theoretical one (559 mAh g^−1^ for Co@C, 373 mAh g^−1^ for Fe@C and 273 mAh g^−1^ for CoFe@C implying the high potential for further improvement. The higher capacity of CoFe@C at 100 mAh g^−1^ than the estimated one remains unclear. The calculations were performed based on the general alloy phase of Co and/or Fe with Al^31–33^ and composition content (41% for Co@C, 26% for Fe@C and 39% for CoFe@C) calculated by referring previously reported methods^9,21^. EIS (Supplementary Fig. S18) of the CoFe@C was measured in the same pouch cell. The EIS curve was studied with an equivalent circuit constructed with internal resistance (*R*_s_, ~50 Ω) including electrolyte resistance, electrode resistance, electrode/current collector contact resistance, and current collector resistance. Furthermore, charge transfer resistance (*R*_ct_, ~1500 Ω) and Warburg impedance (*Z*_w_) representing the Al_2_Cl_7_^−^/AlCl_4_^−^ diffusion resistance, and double layer capacitance (*C*_1_) at the electrode/electrolyte interface are depicted in the EIS curve.

The charge/discharge mechanisms of Co@C, Fe@C, and CoFe@C were further studied with *ex-situ* XRD (Supplementary Fig. S19), where a single C phase was indexed at both the charge (1.2 V vs. AlCl_4_^−^/Al) and discharge (0.05 V vs. AlCl_4_^−^/Al) states. Afterward, SEM (Supplementary Fig. S20) and TEM (Supplementary Fig. S21) were performed for the electrodes after long-term measurements. The active materials were retained tightly adhered as exhibited in the SEM images. Furthermore, the TEM images of electrodes after long-term cycling measurements show recognizable NPs. However, decreases in crystallinities of the electrode materials are also suggested by the halo rings (Supplementary Fig. S21d–f). We thus may be able to preliminarily pursue the possible electrochemical reactions to be xAl^3+^ + 3xe^−^ + Co ↔ Al_x_Co for Co@C and yAl^3+^  + 3ye^−^ + Fe ↔ Al_y_Fe for Fe@C similar to other reports^12,22,34^. The dual peaks (cathodic) for Co@C may imply a sequential reactions^35^. Accordingly, the possible electrochemical reaction for CoFe@C might be CoFe + zAl^3+^  + 3ze^−^ ↔ Al_z_CoFe. The difference in the electrochemical reaction potentials between the Co@C or Fe@C and CoFe@C might be explained by the species variation^36–38^. Therefore, we speculated that the underlying mechanism may be a solid-state diffusion-limited ion insertion/extraction process. However, a further in-depth study is required to provide sufficient evidence.

## Conclusion

We herein verified the electrochemical activity of elemental metal NPs as cathode materials for AIBs. The metal NPs were formed by *in-situ* grown PBAs (CoHCCo, FeHCFe, and CoHCFe) on natural and low-cost L surface, followed by carbonization. After heating, a crystallized C wrapping layer formed spontaneously on the surface of the metal NPs. When the formed Co@C, Fe@C, and CoFe@C were used as the cathode materials in AIBs, respectively, the CoFe@C exhibited a superior charge/discharge capacity (372 mAh g^−1^) to others (103 mAh g^−1^ for Co@C and 75 mAh g^−1^ for Fe@C). The metal NPs demonstrated a stable electrochemical process with apparent discharge plateaus. Typically, CoFe@C would demonstrate an extremely long-term charge/discharge cycling with a capacity decay of 0.7% for each cycle and a Coulombic efficiency of 94.1%. The *ex-situ* characterization had allowed us to conclude that the electrochemical activity of the metal NPs toward charge storage was primarily benefitting from a solid-state diffusion-controlled process. This study is expected to contribute toward the realization of low-cost, innovative, and high-performance cathode materials for AIBs.

## Supplementary information

Graphite carbon-encapsulated metal nanoparticles derived from Prussian blue analogs growing on natural loofa as cathode materials for rechargeable aluminum-ion batteries

## Data Availability

The data that support the findings of this study are available from the corresponding authors upon reasonable request.
