# Supplementary material for: Graphite carbon-encapsulated metal nanoparticles derived from Prussian blue analogs growing on natural loofa as cathode materials for rechargeable aluminum-ion batteries
Source: Sci Rep. 2019 Sep 20;9:13665. doi: 10.1038/s41598-019-50154-8 (PMC6754498; doi:10.1038/s41598-019-50154-8)
Supplement: Supplementary file 1 — Graphite carbon-encapsulated metal nanoparticles derived from Prussian blue analogs growing on natural loofa as cathode materials for rechargeable aluminum-ion batteries [file 41598_2019_50154_MOESM1_ESM.docx]

**Supplementary Information**

**Graphite carbon-encapsulated metal nanoparticles derived from Prussian blue analogs growing on natural loofa as cathode materials for rechargeable aluminum-ion batteries**

Kaiqiang Zhang^1,2^, Tae Hyung Lee^1^, Bailey Bubach^3^, Ho Won Jang^1^, Mehdi Ostadhassan^3*^, Ji-Won Choi^2*^ & Mohammadreza Shokouhimehr^1,3*^

^1^Department of Materials Science and Engineering, Research Institute of Advanced Materials, Seoul National University, Seoul 08826, Republic of Korea.

^2^Electronic Materials Center, Korea Institute of Science and Technology (KIST), Seoul 136-791, South Korea.

^3^Department of Petroleum Engineering, University of North Dakota, Grand Forks, ND 58202, United States.

* Corresponding authors.

E-mail address: Mehdi.Ostadhassan@und.edu (M. Ostadhassan), jwchoi@kist.re.kr (J.-W. Choi), mrsh2@snu.ac.kr (M. Shokouhimehr)


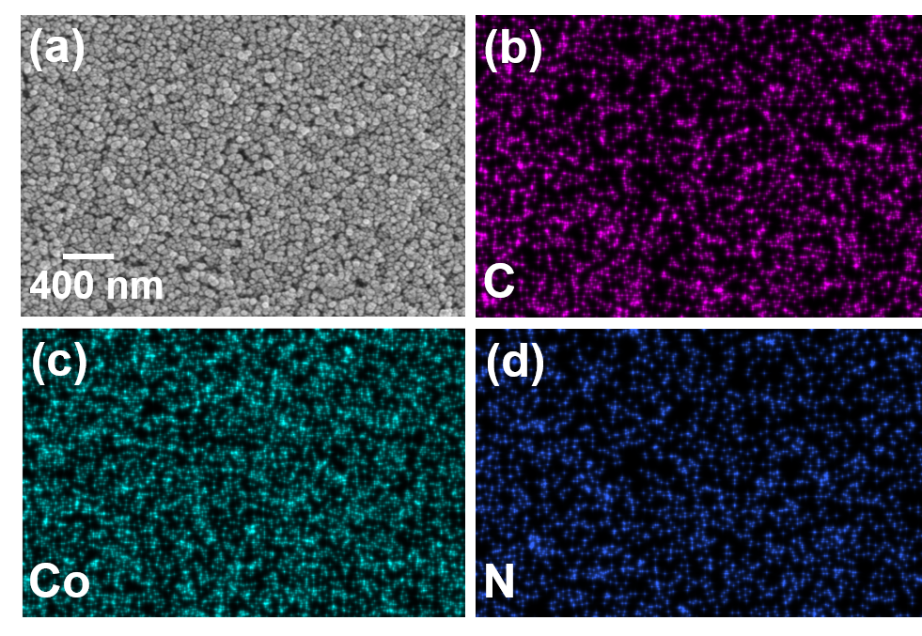


**Figure S1.** EDX mapping of the synthesized CoHCCo powders exhibiting the uniformly distributed elements (C, N and Co).


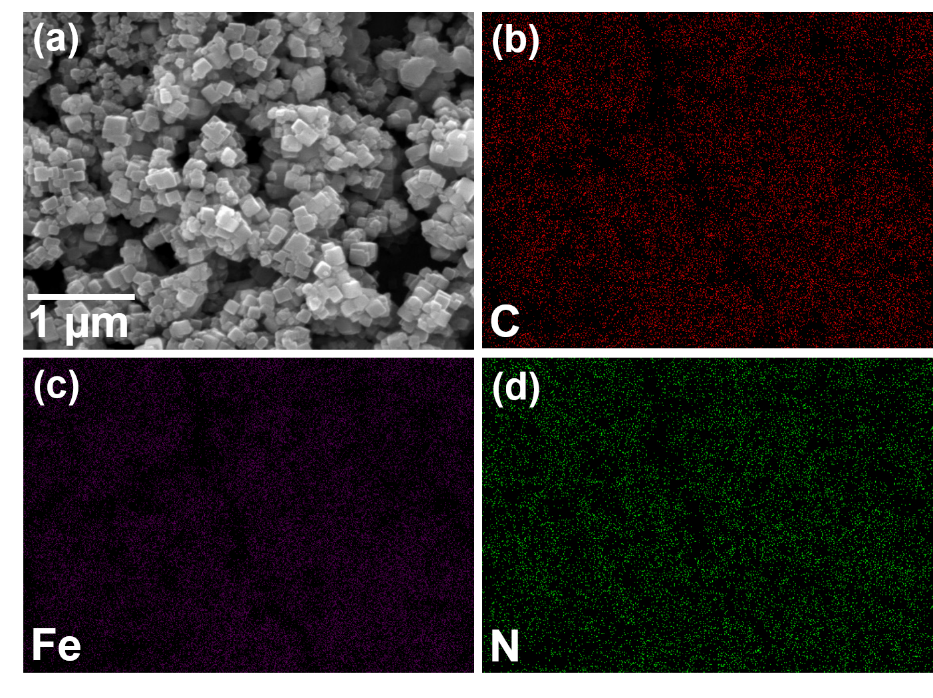


**Figure S2.** EDX mapping of the synthesized FeHCFe powders exhibiting the uniformly distributed elements (C, N and Fe).


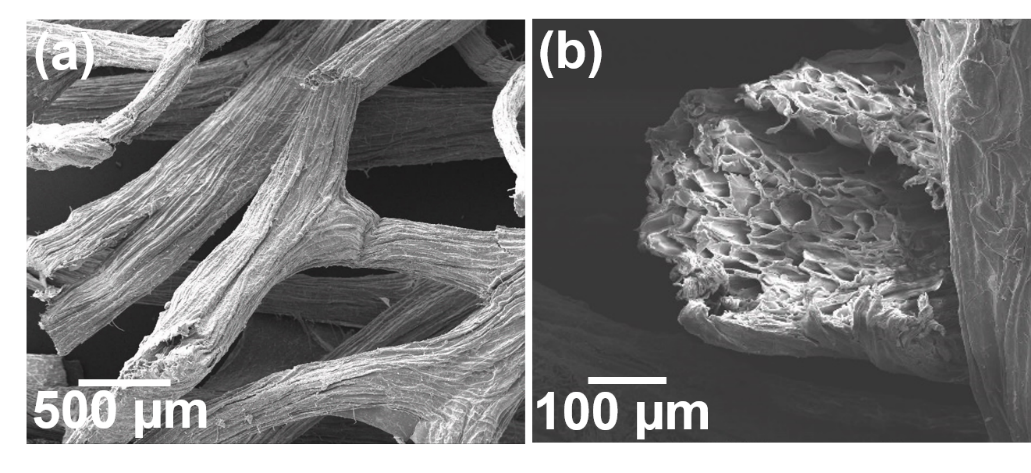


**Figure S3.** (**a**) SEM image of the original loofa, i.e. a ribbon of three dimensional structure. (**b**) SEM image of the cross section of the original loofa ribbon displaying a natural porous morphology.


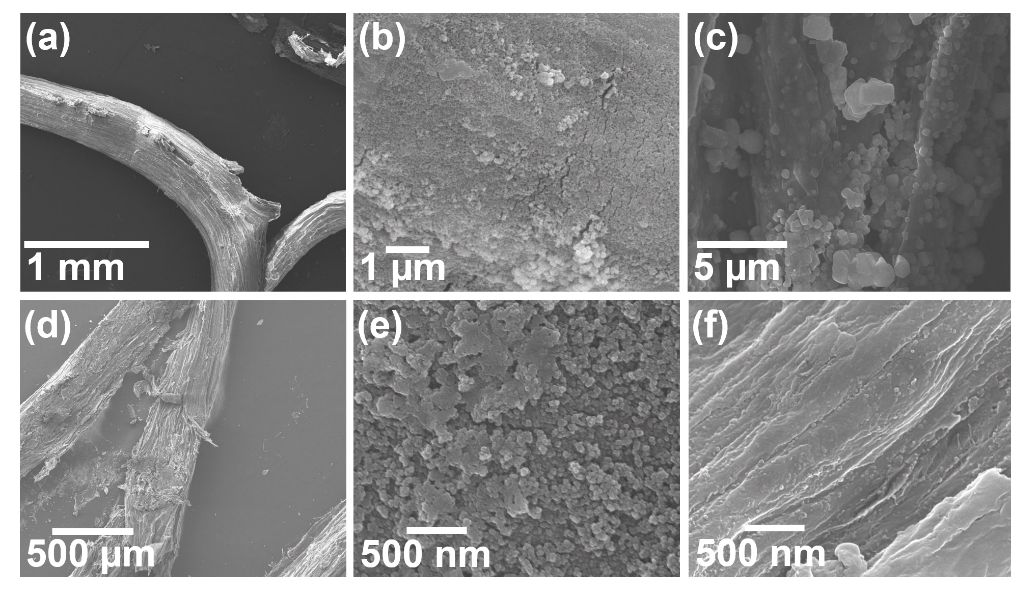


**Figure S4.** SEM images of the (**a**) CoHCCo-loaded loofa, (**d**) FeHCFe-loaded loofa. We deliberately pulverized the loofa ribbon to ensure that its NPs are loaded inside. SEM images of loofa ribbon outer surface loaded with (**b**) CoHCCo and (**e**) FeHCFe exhibiting a large amount of loading. SEM images of loofa ribbon inner surface loaded with (**c**) CoHHCo and (**f**) FeHCFe displaying a successful deposition of PBAs on the inner surface, although a relatively small loading amount was observed on the inner surface than in the outer surface by the SEM images.


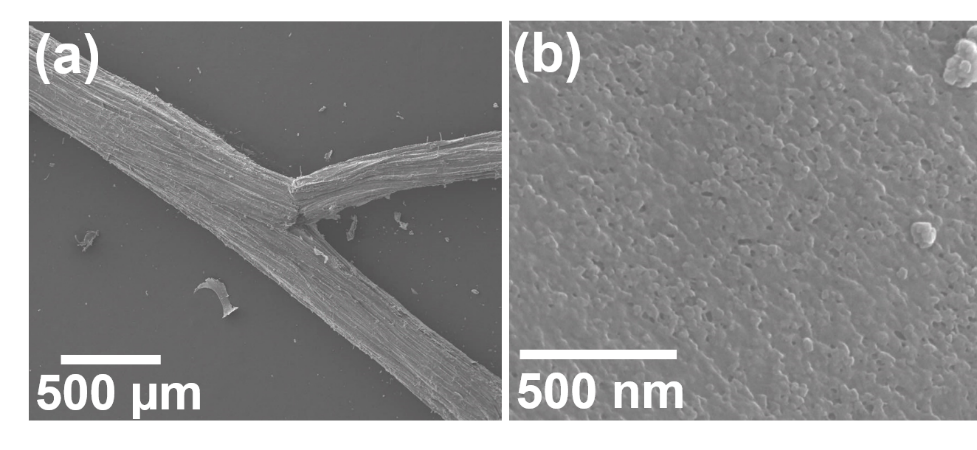


**Figure S5.** SEM images of (**a**) the deliberately teared loofa ribbon, and (**b**) the inner surface of loofa ribbon exhibiting a dense loading of CoHCFe by morphology.


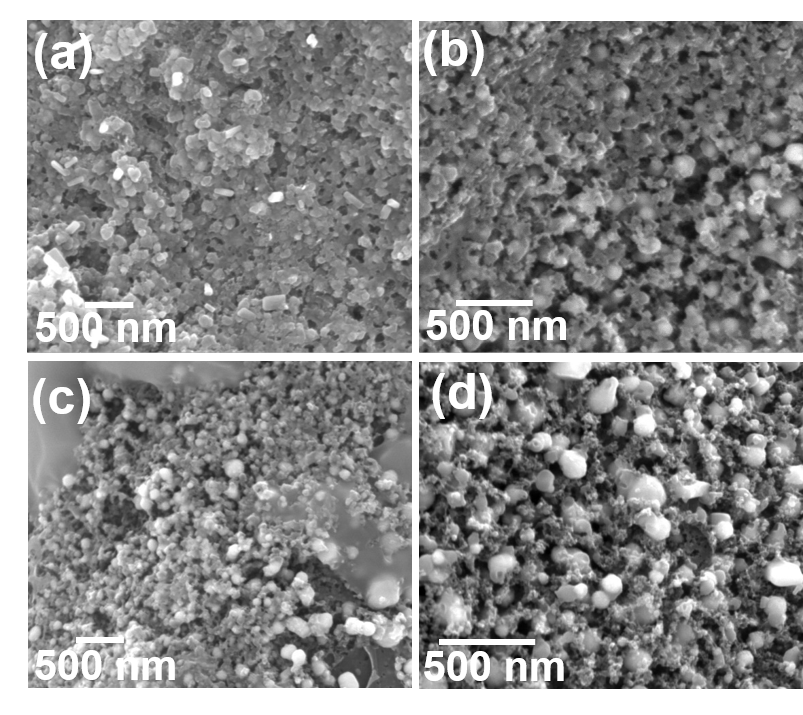


**Figure S6.** SEM images of carbonized CoHCCo/L at various conditions **(a**) 700 °C, 1 h; (**b**) 700 °C, 5 h; (**c**) 900 °C, 1 h; (**d**) 900 °C, 5 h.


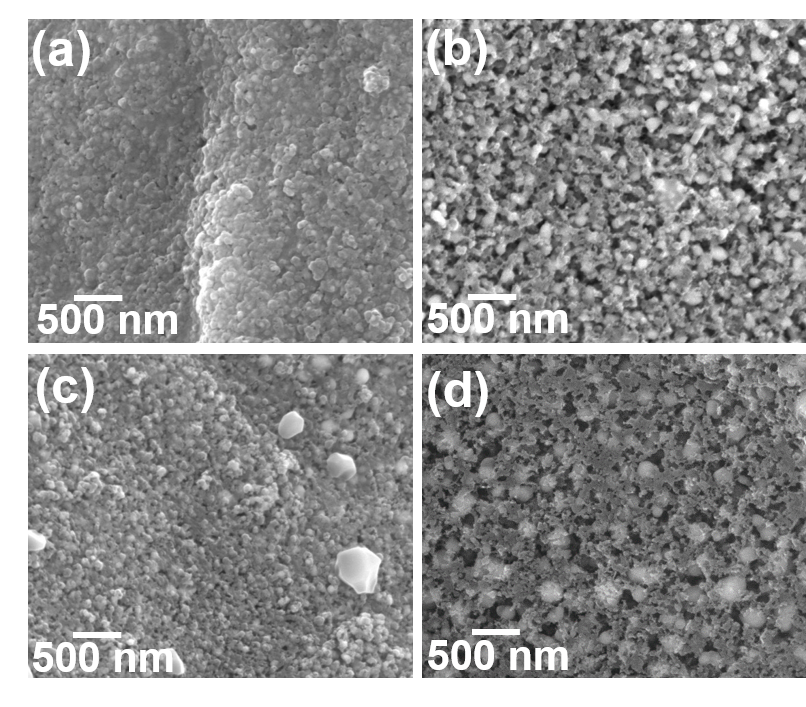


**Figure S7.** SEM images of carbonized FeHCFe/L at various conditions (**a**) 700 °C, 1 h; (**b**) 700 °C, 5 h; (**c**) 900 °C, 1 h; (**d**) 900 °C, 5 h.


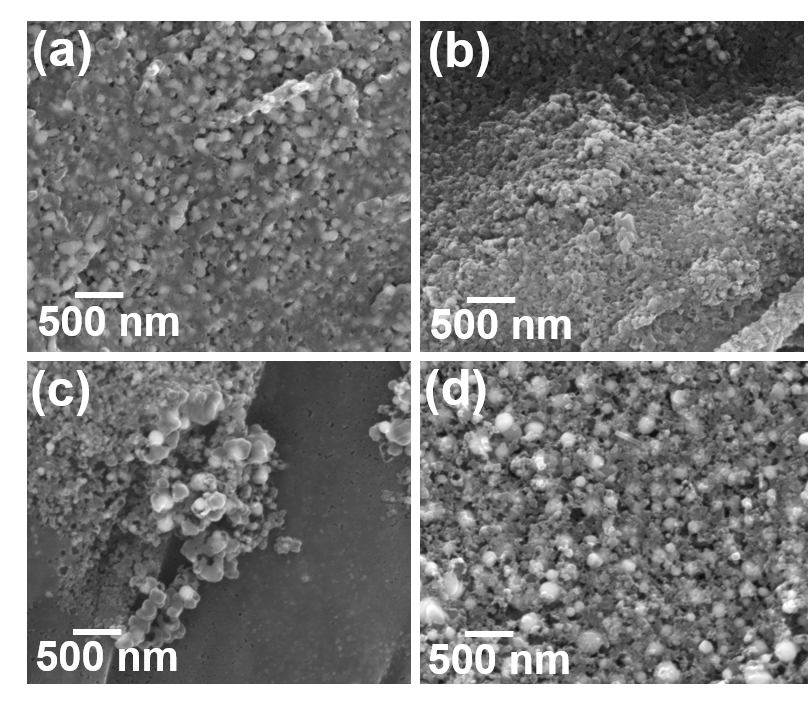


**Figure S8.** SEM images of carbonized CoHCFe/L at various conditions (**a**) 700 °C, 1 h; (**b**) 700 °C, 5 h; (**c**) 900 °C, 1 h; (**d**) 900 °C, 5 h.


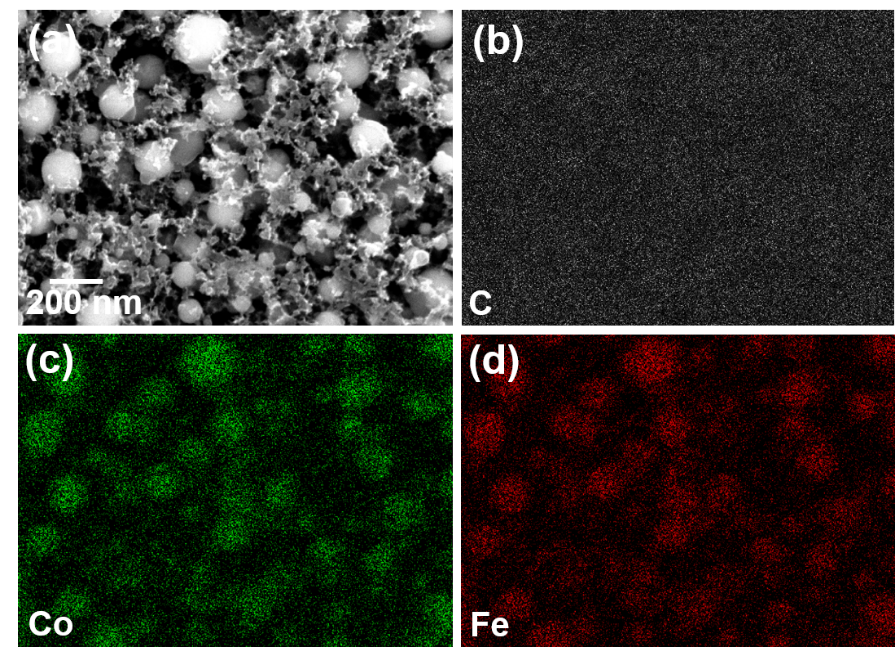


**Figure S9.** SEM image and EDX mapping of CoFe@C.


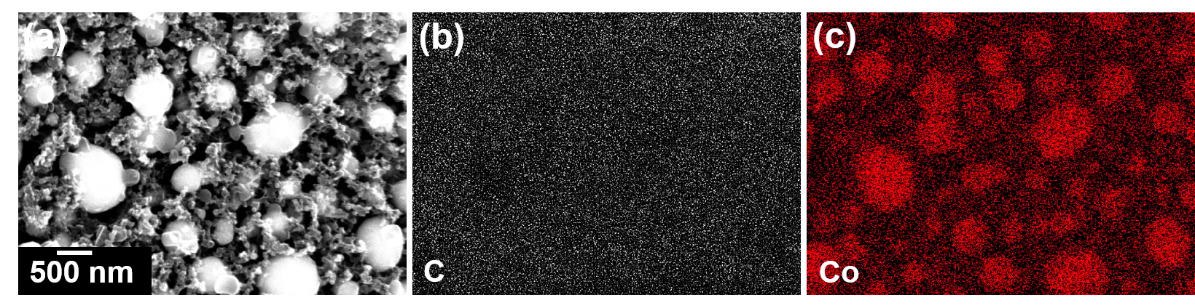


**Figure S10.** SEM image and EDX mapping of Co@C.


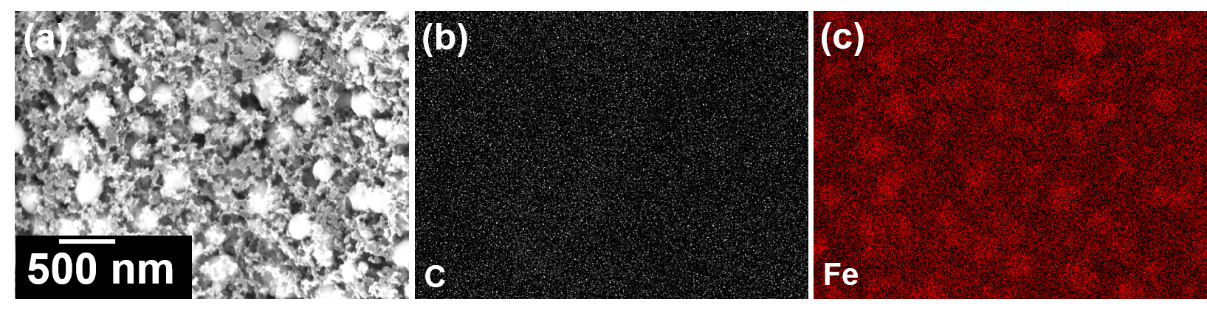


**Figure S11.** SEM image and EDX mapping of Fe@C.


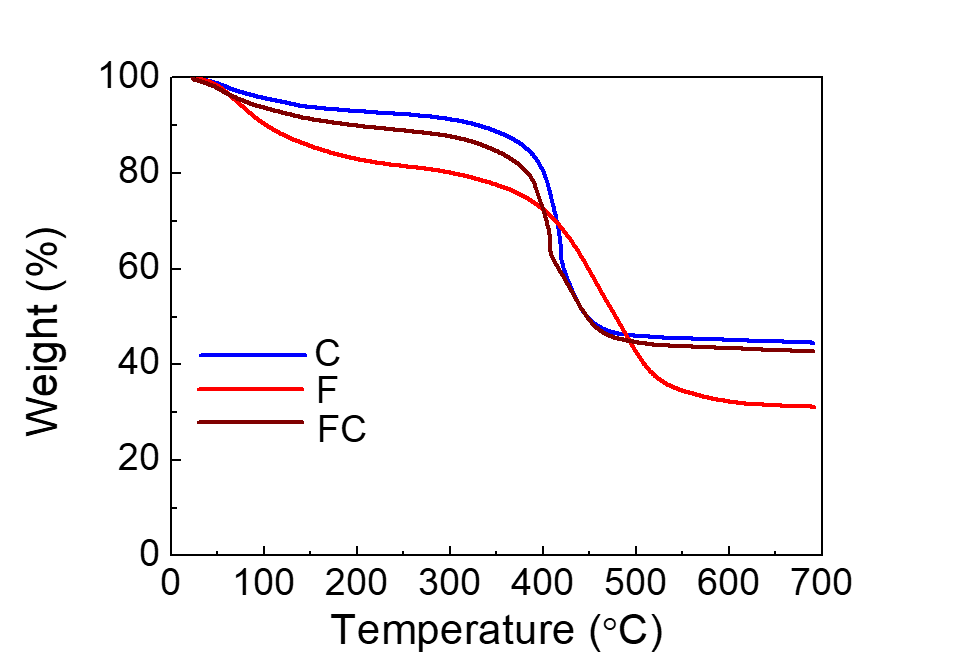


**Figure S12.** TGA curves of Co@C, Fe@C, and CoFe@C.


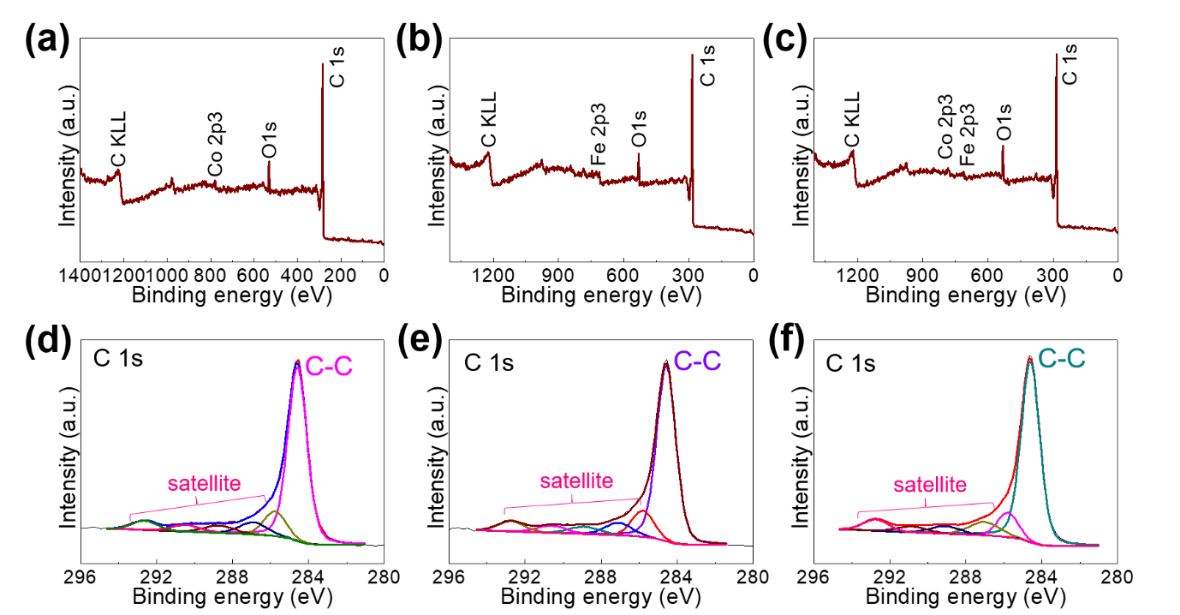


**Figure S13.** XPS spectra of (**a**,**d**) Co@C, (**b**,**e**) Fe@C, and (**c**,**f**) CoFe@C.


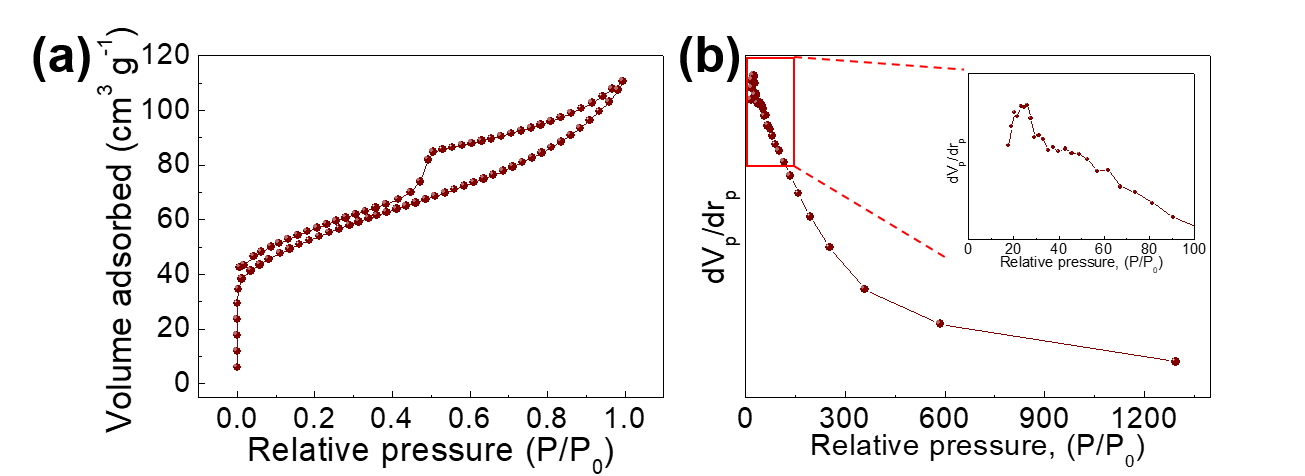


**Figure S14.** (**a**) N_2_ adsorption–desorption isotherms and (**b**) pore size distribution of CoFe@C.


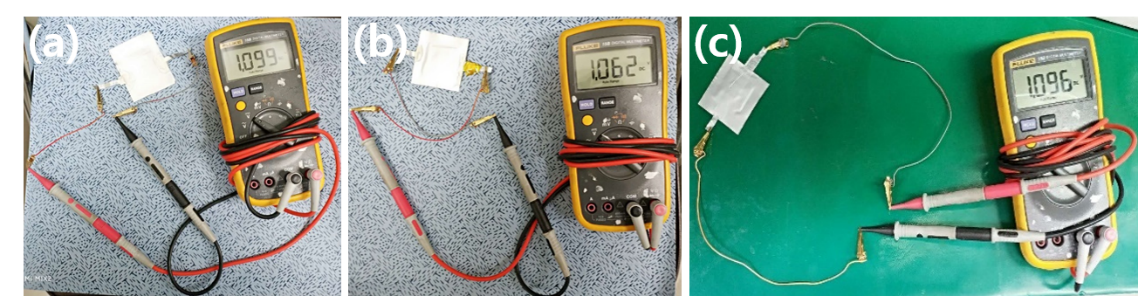


**Figure S15.** OCV of assembled pouch cells with (**a**) Co@C, (**b**) Fe@C, and (**c**) CoFe@C as cathode materials.


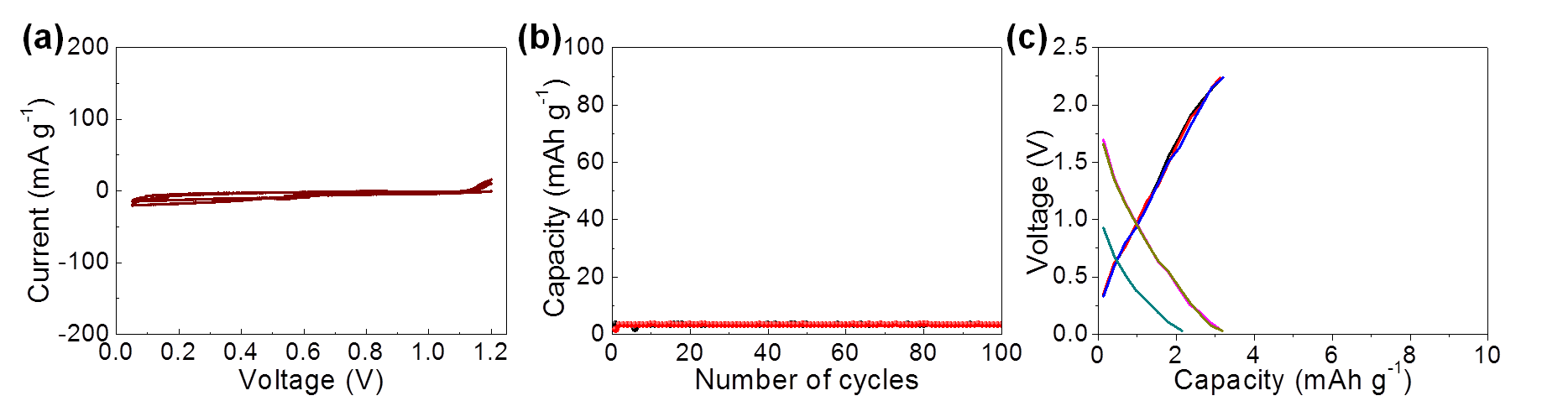


**Figure S16.** (**a**) CV curves of C derived from naked L, (**b**) repeated charge/discharge cycling measurement of C at current density 100 mA g^-1^, and (**c**) typical voltage profiles of C demonstrating a slight capacitive process.


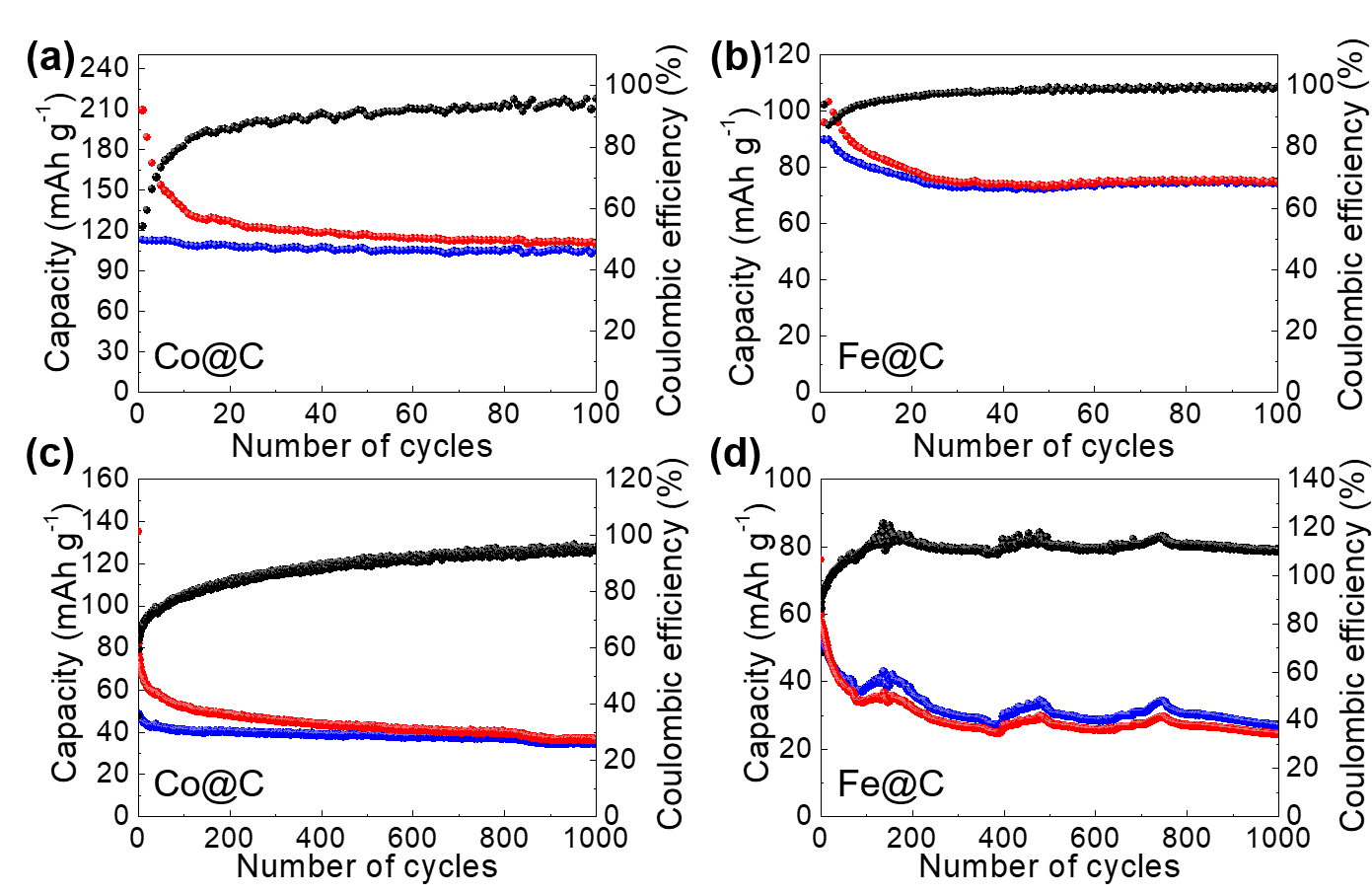


**Figure S17.** Repeated charge/discharge cycling test at 100 mA g^-1^ for (**a**) Co@C and (**b**) Fe@C and at 1,000 mA g^-1^ for (**c**) Co@C and (**d**) Fe@C.


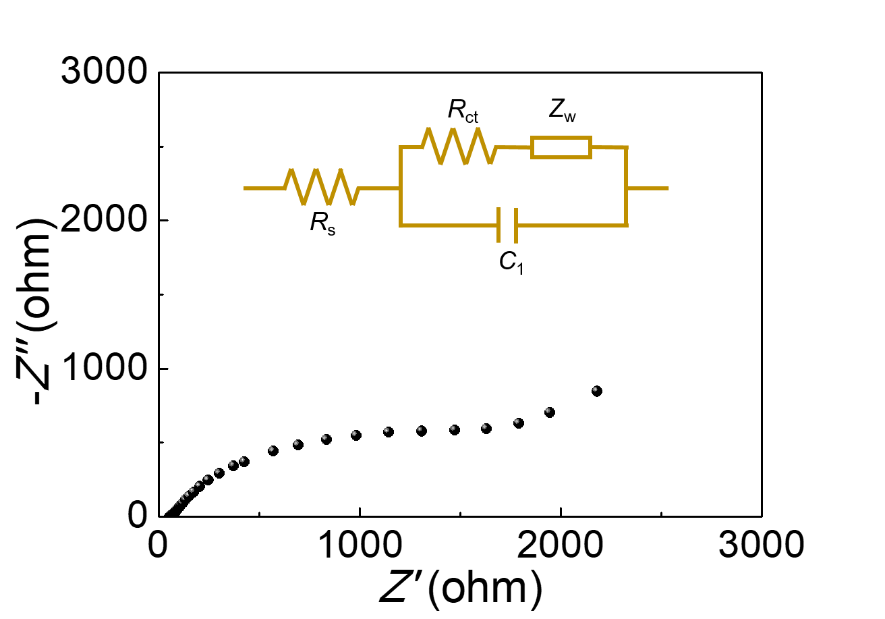


**Figure S18.** EIS spectra of CoFe@C electrode material.


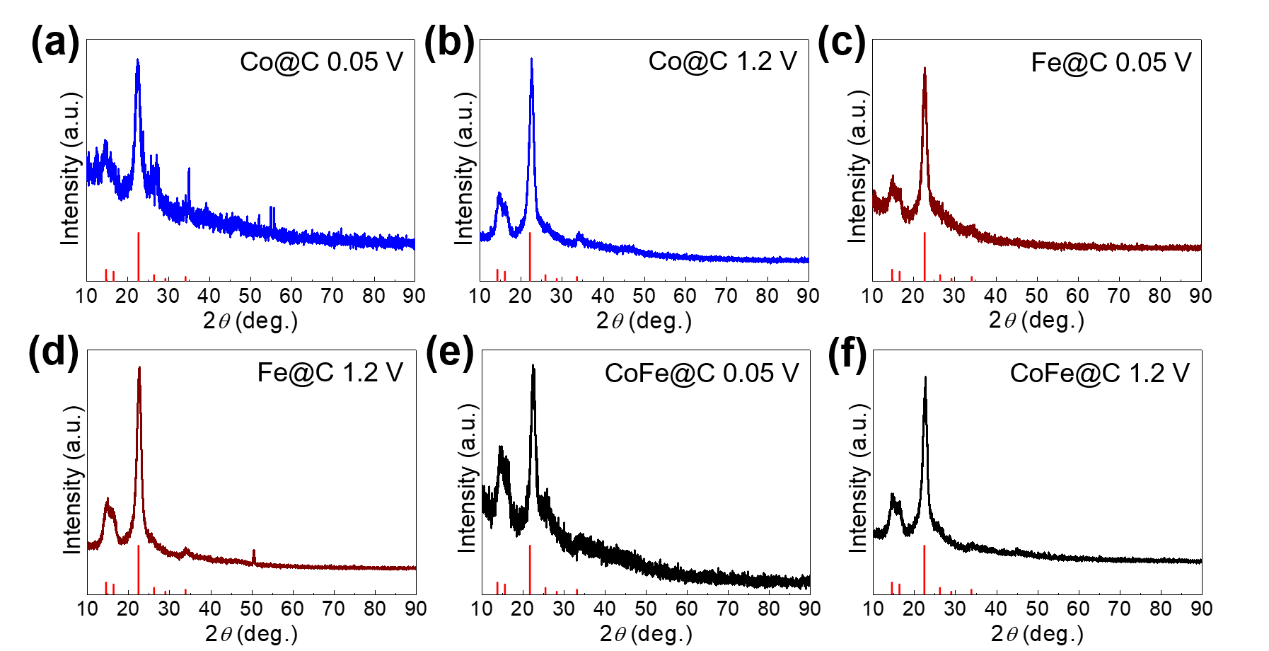


**Figure S19.** Ex-situ XRD results of (**a**,**b**) Co@C, (**c**,**d**) Fe@C, and (**e**,**f**) CoFe@C at different voltages.


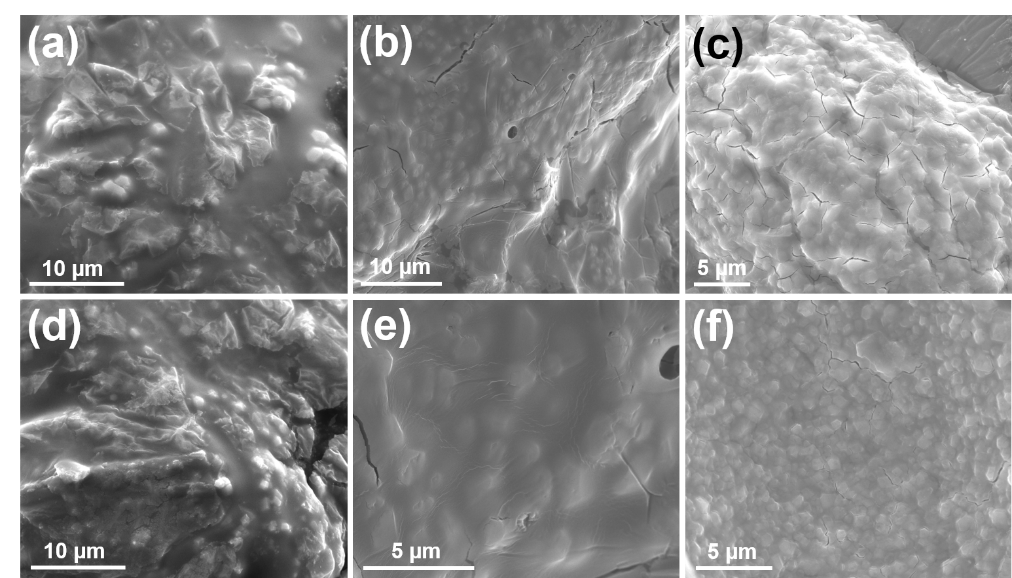


**Figure S20.** SEM images of the (**a**,**d**) Co@C, (**b**,**e**) Fe@C and (**c**,**f**) CoFe@C after long-term repeated charge/discharge cycling measurements at a current density of 1,000 mA g^-1^.


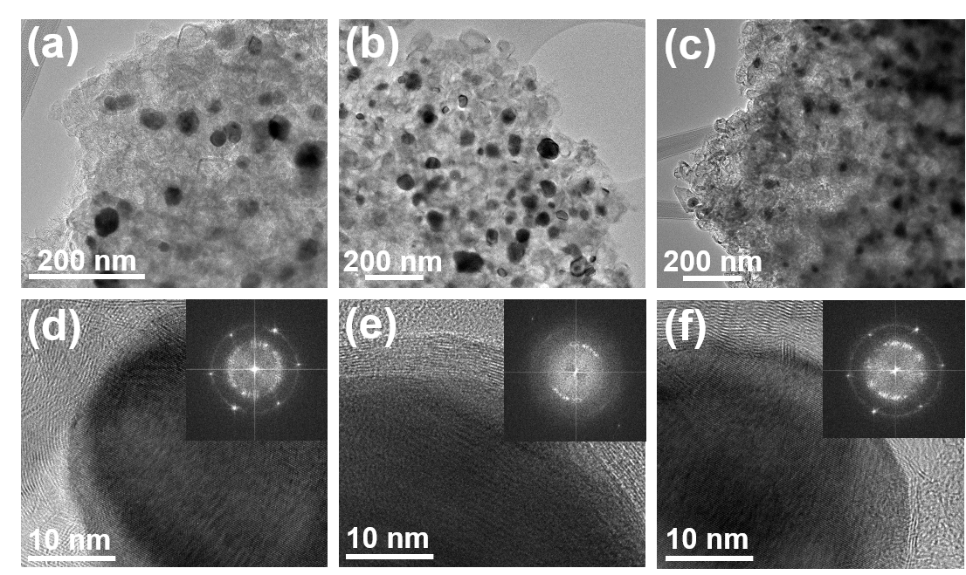


**Figure S21.** TEM images of the (**a**,**d**) Co@C, (**b**,**e**) Fe@C and (**c**,**f**) CoFe@C after long-term repeated charge/discharge cycling measurements at a current density of 1,000 mA g^-1^.
